# Salvage Stereotactic Body Radiotherapy for Locally Recurrent Non-Small Cell Lung Cancer after Sublobar Resection and I^125^ Vicryl Mesh Brachytherapy

**DOI:** 10.3389/fonc.2015.00109

**Published:** 2015-05-11

**Authors:** Beant S. Gill, David A. Clump, Steven A. Burton, Neil A. Christie, Matthew J. Schuchert, Dwight E. Heron

**Affiliations:** ^1^Department of Radiation Oncology, University of Pittsburgh Cancer Institute, Pittsburgh, PA, USA; ^2^Department of Cardiothoracic Surgery, University of Pittsburgh Cancer Institute, Pittsburgh, PA, USA

**Keywords:** SBRT, radiosurgery, re-irradiation, lung cancer, brachytherapy, non-small cell lung cancer, recurrent

## Abstract

**Purpose:**

Locally recurrent non-small cell lung cancer (LR-NSCLC) remains challenging to treat, particularly in patients having received prior radiotherapy. Heterogeneous populations and varied treatment intent in existing literature result in significant limitations in evaluating efficacy of lung re-irradiation. In order to better establish the impact of re-irradiation in patients with LR-NSCLC following high-dose radiotherapy, we report outcomes for patients treated with prior sublobar resection and brachytherapy that subsequently underwent stereotactic body radiotherapy (SBRT).

**Methods:**

A retrospective review of patients initially treated with sublobar resection and I^125^ vicryl mesh brachytherapy, who later developed LR-NSCLC along the suture line, was performed. Patients received salvage SBRT with curative intent. Dose and fractionation were based on tumor location and size, with a median prescription dose of 48 Gy in 4 fractions (range 20–60 Gy in 1–4 fractions).

**Results:**

Thirteen consecutive patients were identified with median follow-up of 2.1 years (range 0.7–5.6 years). Two in-field local failures occurred at 7.5 and 11.1 months, resulting in 2-year local control of 83.9% (95% CI, 63.5–100.0%). Two-year disease-free survival and overall survival estimates were 38.5% (95% CI, 0.0–65.0%) and 65.8% (95% CI, 38.2–93.4%). Four patients (31%) remained disease-free at last follow-up. All but one patient who experienced disease recurrence developed isolated or synchronous distant metastases. Only one patient (7.7%) developed grade ≥3 toxicity, consisting of grade 3 esophageal stricture following a centrally located recurrence previously treated with radiofrequency ablation.

**Conclusion:**

Despite high-local radiation doses delivered to lung parenchyma previously with I^125^ brachytherapy, re-irradiation with SBRT for LR-NSCLC results in excellent local control with limited morbidity, allowing for potential disease cure in a subset of patients.

## Introduction

Improved access to computed tomography (CT) and adoption of screening with low-dose CT, which has been proven to reduce lung cancer mortality, has led to greater detection of earlier stage lung cancers in a high-risk population (1, 2). Despite this improvement in screening, approximately 25% of patients with early stage non-small cell lung cancer (NSCLC) have poor pulmonary function, limiting their ability to tolerate lobectomy (3). To avoid the survival detriment seen with untreated NSCLC, potentially curative alternatives for this medically high-risk population include stereotactic body radiotherapy (SBRT), hypofractionated conventional radiotherapy, radiofrequency ablation, and sublobar resection (4–8). Historical data suggested sublobar resection resulted in inferior local control as compared to lobectomy, leading to integration of I^125^ vicryl mesh brachytherapy to reduce this risk (9, 10).

Recently published results from a randomized trial demonstrate a low rate of local relapse altogether, resulting in no demonstrated benefit to vicryl mesh brachytherapy following sublobar resection (11). Nonetheless, local relapse for patients treated with prior brachytherapy or high-dose radiotherapy such as SBRT has limited salvage options following locally recurrent disease due to concerns of toxicity with lung re-irradiation coupled with poor pulmonary reserve. Without effective salvage therapy, locoregional recurrence often results in death (12, 13). Re-irradiation with SBRT or EBRT has been previously evaluated with varying results regarding both toxicity and clinical outcomes (14–22).

No published data exist regarding treatment of patients following vicryl mesh brachytherapy, where greater concern for necrosis and pneumonitis theoretically may exist due to high-local doses. Here, we report outcomes and toxicities from a subset of patients with locally recurrent NSCLC following sublobar resection and I^125^ vicryl mesh brachytherapy treated with salvage SBRT.

## Materials and Methods

Following Institutional Review Board approval, a retrospective review was conducted for patients with NSCLC treated with SBRT at the University of Pittsburgh Cancer Institute. Patients included previously received sublobar resection with I^125^ vicryl mesh brachytherapy for a primary NSCLC, later developing local recurrence adjacent to the brachytherapy mesh. All patients received re-irradiation using SBRT with varying fractionation regimens, based on the proximity of critical structures and at discretion of the treating physician. Re-irradiation was defined by the relation of the planning target volume (PTV) to the vicryl mesh, such that the PTV was within 1 cm from the mesh.

At the time of recurrence, patients underwent either CT or ^18^F-fluorodeoxyglucose (^18^F-FDG) positron emission tomography/CT (PET/CT) for re-staging and/or radiation treatment planning. Patients with biopsy-confirmed or radiographic nodal or distant metastases were excluded. Determination of recurrent NSCLC was either histologically proven or radiographically defined based on morphology and/or serial imaging.

Patients received SBRT through various platforms: CyberKnife ™ (Accuray, Inc., Sunnyvale, CA, USA), Trilogy ™ (Varian Medical Systems, Palo Alto, CA, USA), or TrueBeam ™ (Varian Medical Systems). Treatment simulation consisted of a four-dimensional high-resolution CT scan (4DCT) with intravenous contrast if medically feasible. A custom BodyFIX ™ vacuum bag (Elekta AB, Stockholm, Sweden) was used for immobilization. Respiratory gating was then utilized for TrueBeam ™ or Trilogy ™ treatment based on tumor motion, with a cut-off of >5 mm in any dimension on raw phase images to indicate the need for gating. The Synchrony ™ Respiratory Tracking System (Accuray, Inc.) was utilized for real-time tracking with CyberKnife ™, in conjunction with pre-placed fiducials.

Treatment planning in either MultiPLAN ™ (Accuray, Inc.) or Eclipse (Varian Medical Systems) was completed, identifying the gross tumor volume (GTV) on end-exhalation or free breathing CT simulation scans based on the need for gating. Tumors treated on the CyberKnife ™ platform had PTV expansions of 1 cm in the craniocaudal direction and 0.5 cm radially similar to that in Radiation Therapy Oncology Group (RTOG) 0236 (4). For TrueBeam ™ or Trilogy ™ treatment, a minimum expansion of 5 mm was added for a PTV, incorporating an additional margin for tumor motion assessed on 4DCT. Typically, an incorporated internal target volume (ITV) involved adding the extent of motion within the gated window to the minimum PTV margin in the direction of movement (23). Given variations in fractionation regimens, dosimetric constraints varied although at least 95% of the PTV was expected to be covered by the prescription dose. Treatment was delivered every other day.

Follow-up imaging consisted of CT or PET/CT at intervals based on physician discretion, initially starting 8–12 weeks from completion of SBRT. Criteria for local failure were based on the RTOG 0236 definition: ≥20% increase in greatest dimension per CT and evidence of tumor viability via FDG-avidity or histologic confirmation (4). Regional failure included hilar, mediastinal, and/or supraclavicular nodal failure. All other failures, including the contralateral lung, were coded as distant metastases unless a new solitary lung lesion was present, suggestive of a new primary lung cancer. Common Terminology Criteria for Adverse Events (version 4.03) was used to record toxicity.

Statistical analysis was conducted using SPSS version 22 (IBM Corp., Armonk, NY, USA). Kaplan–Meier methods were used to assess local control, distant-metastasis free survival, disease-free survival, and overall survival. Log-rank test was conducted to assess factors associated with the various treatment outcomes. Biological effective doses (BEDs) were calculated using the linear-quadratic equation with an α/β value of 10 for tumor. For descriptive purposes, BED values were converted to equivalent dose at 2 Gy (EQD_2_) when discussing toxicity.

## Results

Thirteen patients were identified with recurrent NSCLC along the brachytherapy mesh, of which nine patients (69%) had histologic confirmation (Table 1). Recurrence occurred at a median of 3.8 years from initial diagnosis (range 0.9–9.5 years). Despite a median age of 71 years, the median Karnofsky performance status score was 90% (range 60–100%). The right upper (38%) and left upper (31%) lobes were the most common locations, with most recurrences located >2 cm from the central bronchial tree (69%). Patients were treated using either TrueBeam/Trilogy (46%) or CyberKnife (54%). The most common fractionation schemes were 48 Gy in 4 fractions (46%) or 60 Gy in 3 fractions (38%), resulting in a median BED_10_ of 105.6 Gy (Table 2).

**Table 1:** **Patient and disease-related characteristics at the time of stereotactic body re-irradiation**.

	Value
**Age, median (range)**	71 years (54–87 years)
**KPS, median (range)**	90% (60–100%)
**Gender (*n*, %)**	
Male	7 (54%)
Female	6 (46%)
**History of tobacco smoking (*n*, %)**	
Yes	13 (100%)
No	0 (0%)
**Initial AJCC T stage (*n*, %)**	
T1a–b	5 (38.5%)
T2a–b	5 (38.5%)
T3	1 (8%)
Unknown	2 (15%)
**Prior therapy following recurrence (*n*, %)**	
Radiofrequency ablation	3 (23%)
None	10 (77%)
**Histology (*n*, %)**	
Squamous cell carcinoma	5 (38.5%)
Adenocarcinoma	7 (53.5%)
Non-small cell carcinoma, NOS	1 (8%)
**Time to recurrence, median (range)**	3.8 years (0.9–9.5 years)
**Diagnostic criteria for recurrence (*n*,%)**	
Biopsy-proven	9 (69%)
Clinical/radiographic	4 (31%)
**Location (lobe) of recurrence (*n*, %)**	
Right upper lobe	5 (38%)
Right middle lobe	0 (0%)
Right lower lobe	3 (23%)
Left upper lobe	4 (31%)
Left lower lobe	1 (8%)
**Location of recurrence (*n*, %)**	
Central	4 (31%)
Peripheral	9 (69%)

*KPS, Karnofsky performance status; AJCC, American Joint Committee on Cancer; NOS, not otherwise specified*.

**Table 2:** **Stereotactic body radiotherapy (SBRT) re-irradiation characteristics**.

	All patients (*n* ***=*** 13)	TrueBeam/Trilogy (*n* ***=*** 6)	CyberKnife (*n* ***=*** 7)
**PTV volume**			
Median (range)	25.3 cc (10.8–107.8 cc)	26.8 cc (10.8–107.8 cc)	25.3 cc (14.7–52.6 cc)
**Number of non-zero beams/fields**			
Median (range)	–	11 (10–12)	154 (137–162)
**Dose-fractionation schedule (*n*, %)**			
9 Gy × 5 fractions	1 (8%)	1 (17%)	0 (0%)
12 Gy × 4 fractions	6 (46%)	3 (50%)	3 (43%)
20 Gy × 3 fractions	5 (38%)	2 (33%)	3 (43%)
20 Gy × 1 fraction	1 (8%)	0 (0%)	1 (14%)
**Re-irradiation BED_10_**			
Median (range)	105.6 Gy (60.0–180.0 Gy)	105.6 Gy (85.5–180.0 Gy)	105.6 Gy (60.0–180.0 Gy)
**Prescription isodose line**			
Median (range)	80% (80–90%)	86% (82–90%)	80% (80–80%)
**Minimum PTV dose, relative to prescription dose**			
Median (range)	83% (50–100%)	89% (75–100%)	66% (50–90%)
**Heterogeneity index**			
Median (range)	1.23 (1.10–1.25)	1.15 (1.10–1.22)	1.25 (1.23–1.25)
**Median *R_50%_***
All PTVs	3.9	5.2	3.0
PTV <20 cc	4.3	4.8	3.8
PTV 20–50 cc	3.0	5.5	2.4
PTV >50 cc	4.7	6.4	2.9
**Treatment time**			
Median (range)	13 days (1–16 days)	12.5 days (5–16 days)	9 days (1–13 days)

*SBRT, stereotactic body radiotherapy; PTV, planning target volume; BED, biological effective dose; *R*_50%_, ratio of 50% prescription isodose volume to the PTV*.

**Figure 1:**
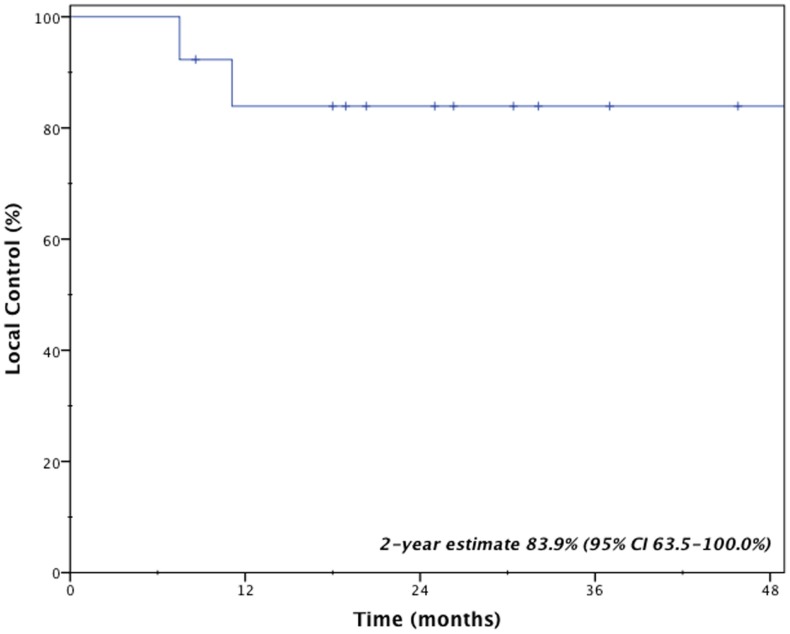
**Kaplan–Meier estimate of local control**.

Clinical outcomes are indicated in Table 3. With a median follow-up time of 2.1 years (range 0.7–5.6 years), two patients (15.4%) developed local failure, one with isolated local failure and the other patient with simultaneous local, regional, and distant failure. Both local recurrences occurred within the planning target volumes at 7.5 and 11.1 months after receiving a BED_10_ of 85.5 and 180.0 Gy, respectively. Re-irradiation planning treatment volumes for these two patients were 16.6 and 25.3 cc. The 2-year Kaplan–Meier estimated local control rate was 83.9% (95% CI, 63.5–100.0%; Figure 1). No factors were found to be associated with local control, including PTV volume, BED_10_, time to recurrence or tumor location.

**Table 3:** **Clinical outcomes for patients (*n* = 13) treated with SBRT re-irradiation**.

ID	Time to recurrence (years)	Biopsy-proven recurrence	Recurrence location	BED_10_ (Gy)	Last follow-up or death (years)	Disease-free?	Type of failure	Death
1	5.2	Y	Peripheral	60.0	0.7	N	DF	Y
2	1.6	Y	Peripheral	105.6	1.7	N	DF	Y
3	7.3	Y	Peripheral	180.0	2.2	N	RF + DF	Y
4	1.3	N	Peripheral	180.0	1.1	N	LF + RF + DF	Y
5	3.8	N	Peripheral	180.0	2.1	N	DF	Y
6	0.9	Y	Central	105.6	5.6	Y	–	N
7	7.6	Y	Peripheral	105.6	2.5	Y	–	Y
8	2.6	N	Peripheral	180.0	2.7	Y	–	N
9	2.2	Y	Peripheral	105.6	1.6	N	RF + DF	Y
10	4.3	Y	Central	105.6	3.8	N	DF	N
11	9.5	Y	Central	180.0	3.1	Y	–	N
12	2.9	N	Central	85.5	1.5	N	LF	N
13	6.9	Y	Peripheral	105.6	1.5	N	DF	N

*ID, patient number; BED, biologically equivalent dose; LF, local failure; RF, regional failure; DF, distant failure*.

**Figure 2:**
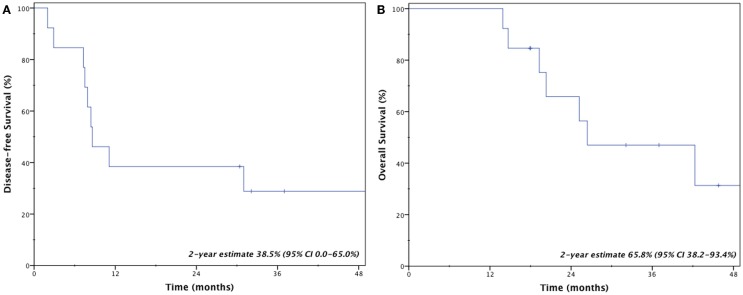
**Kaplan–Meier estimates of disease-free (A) and overall (B) survival**.

Four patients (31%) remain disease-free at last follow-up; three patients (23%) are both alive and disease-free. Crude rates of disease recurrence were as follows: isolated local (*n* = 1, 7.7%); synchronous local, regional, and distant (*n* = 1, 7.7%); synchronous regional and distant (*n* = 2, 15.4%); and isolated distant (*n* = 5, 38.5%). Two-year estimates for disease-free survival and overall survival were 38.5% (95% CI, 0.0–65.0%) and 65.8% (95% CI, 38.2–93.4%), respectively (Figure 2). Median overall survival was 26.4 months.

No patients developed grade 3 or greater pulmonary toxicity, including lung fibrosis and pneumonitis. However, two patients (15.4%) did develop grade 2 fibrosis and grade 2 dyspnea at 9.4 and 10.1 months after treatment. No grade 2 esophageal toxicities were seen. One patient (7.7%) developed a grade 3 esophageal stricture at 3.1 months after treatment requiring endoscopic dilatation. Of particular note, this patient had a central tumor recurrence treated with radiofrequency ablation prior to SBRT. Given proximity to central structures, the patient received 45 Gy in 5 fractions, although the maximal point dose to the esophagus was 38.8 Gy (7.8 Gy/fraction), resulting in an EQD_2_ of 83.8 Gy.

## Discussion

Management of locally recurrent non-small cell lung cancer (LR-NSCLC) remains challenging due to limitations from prior therapy and presence of medical comorbidities that often preclude aggressive therapy. For this reason, less invasive therapies with limited risk of morbidity are often ideal. Stereotactic body radiotherapy provides such benefits and enables the ability to deliver conformal and high doses to tumors. However, for patients who received prior high-dose radiotherapy, concern always exists regarding added toxicity from re-irradiation. Results presented here suggest that even with previous high-local doses to normal lung from brachytherapy, salvage SBRT resulted in limited toxicity and provided an efficacious salvage option for locally recurrent lung cancer. Specifically, the 2-year local control rate remained high at 83.9% with a median survival of 26.4 months.

Clinical outcomes following re-irradiation of LR-NSCLCs have been difficult to interpret due to heterogeneous populations and loose definitions of re-irradiation. For example, among 11 studies reviewed by Jeremic et al., only 3 trials delivered external beam re-irradiation using curative doses (median dose ≥50 Gy) (15). Nonetheless, local control remains limited with external beam re-irradiation, ranging from 16.7 to 42.0% in these trials (14, 24, 25). In a recently published larger cohort of 102 patients, McAvoy et al. identified 41 patients with locoregional recurrence within the prior radiotherapy field, among which 46% local control was achieved after re-irradiation using various modalities (26). Although many of these series included more advanced lung cancer at recurrence, re-irradiation with conventional fractionation appears to result in, at best, modest rates of local control.

Several publications have addressed feasibility and toxicity using SBRT re-irradiation for lung tumors. Many of these series are limited again by mixed treatment intent, varying definitions of re-irradiation and diverse histology and disease stage (16, 17, 19–22). Adequate estimation of long-term clinical outcomes for patients with LR-NSCLC alone within the prior radiotherapy field is therefore difficult to determine. Only two of these studies either reported separate outcomes or included only LR-NSCLC with SBRT re-irradiation defined as overlap with the prior treatment field (17, 19). Hearn et al. reported 10 patients treated with salvage SBRT, resulting in crude local control and overall survival rates of 60 and 30% (17). Parks et al. identified 29 patients treated with repeat SBRT, where 13 patients underwent re-irradiation of in-field recurrences leading to a 2-year locoregional relapse-free survival rate of 58% (19). These two studies suggest that despite a high-equivalent dose delivered using SBRT, locoregional control appears only slightly improved, if not comparable, to other radiotherapy methods. Conversely, in the present study, 2-year local control remained excellent at 83.9%. Such a finding may reflect rigorously selected patients, where many underwent PET/CT re-staging with identification of isolated local disease. Other explanations include comparably long re-treatment intervals (median time to re-irradiation 3.8 years), which may attest to disease biology and initial disease stage. Multivariate analysis of the prior study by McAvoy et al. illustrated improved local control and survival with a re-treatment interval >6 months and lower initial T stage (26). Lastly, in patients with prior brachytherapy, cell-kill mechanisms may be different from that delivered through SBRT. Thus, patients treated with prior brachytherapy may be responsive to re-irradiation using high doses per fraction (i.e., SBRT). Nonetheless, these findings, among a much more homogenous population, should indicate that in properly selected patients, re-irradiation with SBRT for locally recurrent NSCLC can provide improved local control in a shorter treatment course.

Re-irradiation, particularly using high-dose regimens such as that seen with SBRT, comes with added concerns of toxicity. In re-irradiation series using external beam radiotherapy, rates of grade 3 or greater pneumonitis and esophagitis range from 5 to 21 and 4 to 6%, respectively (15). Here, we reported selectively on patients with recurrence near brachytherapy mesh to illustrate that despite prior high-radiation doses, severe pulmonary toxicity rates remain exceedingly low in a carefully planned and well-executed schema of stereotactic radiotherapy, a more conformal technique. Lung parenchyma functions as a parallel organ and thus volume of functional lung irradiated plays a larger factor than maximum point dose. Such findings have been confirmed using external beam radiotherapy, showing that volume of lung irradiated, even at low doses, correlates with risk of pneumonitis and atelectasis (27–30). Similar dose–volume parameters have been established for stereotactic body radiotherapy (31, 32). Utilizing SBRT for re-irradiation of lung lesions limits the volume of normal lung receiving dose greater than that seen with conventional methods, resulting in low rates of severe pneumonitis as confirmed here.

In the setting of SBRT re-irradiation for lung tumors, tumor volume and central structure tolerance should have a greater impact on management decisions as opposed to concerns over high-local doses to lung parenchyma. In our study, we identified one patient who developed late grade 3 esophagitis after receiving adjacent radiofrequency ablation and a maximal point dose of 38.8 Gy (EQD_2_ 83.8 Gy). Studies using external beam radiotherapy for re-irradiation have shown low rates of grade 3 esophagitis (4–6%), although this may be a function of tumor location (15). High doses with SBRT may be less forgiving to central mediastinal structures, as evidenced in both prospective and retrospective series (16, 21, 33). In the setting of re-irradiation, Peulen et al. noted all grade 4–5 toxicities occurred in centrally located lesions (16). Three patients developed grade 5 complications due to hemorrhage. Kilburn et al. noted one patient death due to development of an aortoesophageal fistula (21). Thus, the approach of re-irradiation using SBRT should be taken cautiously for centrally located lesions.

Our study, like many others evaluating re-irradiation, is limited by both the retrospective nature of review and small sample size. We intentionally identified a select population in order to provide a clear analysis of a comparable patient cohort as opposed to that done in a number of re-irradiation studies. Although varying fractionation regimens make direct interpretation challenging, a majority received more commonly utilized regimens (48 Gy in 4 fractions or 60 Gy in 3 fractions). Additionally, with a median follow-up time of 2.1 years, whether these favorable local control rates would persist over time remains unknown. Despite these limitations, these results should provide re-assurance that in properly selected patients with locally recurrent NSCLC, even in heavily irradiated regions, stereotactic body radiotherapy can provide excellent local control with limited morbidity, resulting in cure among a small subset of patients. In the future, better tolerated and/or targeted systemic therapy may aid in decreasing the high rate of distant metastases in this population, which remained the predominant mode of failure.

## Conclusion

Stereotactic body radiotherapy for locally recurrent NSCLC following prior radiotherapy is an effective salvage therapy with limited morbidity, even despite high doses of prior radiotherapy with I^125^ vicryl mesh brachytherapy. Severe pulmonary parenchymal toxicity remains low with re-irradiation using SBRT, likely related to limited dose to large lung volumes. Centrally located tumors should be cautiously selected for re-irradiation using SBRT. Although a proportion of patients may achieve cure, for most patients, optimization of systemic therapy is critical to offset the risk of distant metastases.

## Conflict of Interest Statement

The authors declare that the research was conducted in the absence of any commercial or financial relationships that could be construed as a potential conflict of interest.
